# Bed net use and associated factors in a rice farming community in Central Kenya

**DOI:** 10.1186/1475-2875-8-64

**Published:** 2009-04-16

**Authors:** Peter N Ng'ang'a, Gayathri Jayasinghe, Violet Kimani, Josephat Shililu, Charity Kabutha, Lucy Kabuage, John Githure, Clifford Mutero

**Affiliations:** 1 International Centre of Insect Physiology and Ecology (ICIPE), PO Box 30772, Nairobi, Kenya; 2 International Water Management Institute (IWMI), United Nations Avenue, PO Box 30677-00100, Nairobi, Kenya; 3 Department of Community Health, University of Nairobi, PO Box 29053, Nairobi, Kenya; 4 College of Agriculture and Veterinary Science, University of Nairobi, PO Box 29040, Nairobi, Kenya; 5 International Water Management Institute (IWMI), Private Bag X813, 0127 Silverton, Pretoria, South Africa

## Abstract

**Background:**

Use of insecticide-treated nets (ITNs) continues to offer potential strategy for malaria prevention in endemic areas. However their effectiveness, sustainability and massive scale up remain a factor of socio-economic and cultural variables of the local community which are indispensable during design and implementation stages.

**Methods:**

An ethnographic household survey was conducted in four study villages which were purposefully selected to represent socio-economic and geographical diversity. In total, 400 households were randomly selected from the four study villages. Quantitative and qualitative information of the respondents were collected by use of semi-structured questionnaires and focus group discussions.

**Results:**

Malaria was reported the most frequently occurring disease in the area (93%) and its aetiology was attributed to other non-biomedical causes like stagnant water (16%), and long rains (13%). Factors which significantly caused variation in bed net use were occupant relationship to household head (χ^2 ^= 105.705; df 14; P = 0.000), Age (χ^2 ^= 74.483; df 14; P = 0.000), village (χ^2 ^= 150.325; df 6; P = 0.000), occupation (χ^2 ^= 7.955; df 3; P = 0.047), gender (χ^2 ^= 4.254; df 1; P = 0.039) and education levels of the household head or spouse (χ^2 ^= 33.622; df 6; P = 0.000). The same variables determined access and conditions of bed nets at household level. Protection against mosquito bite (95%) was the main reason cited for using bed nets in most households while protection against malaria came second (54%). Colour, shape and affordability were some of the key potential factors which determined choice, use and acceptance of bed nets in the study area.

**Conclusion:**

The study highlights potential social and economic variables important for effective and sustainable implementation of bed nets-related programmes in Sub-Saharan Africa.

## Background

Treatment, prevention and control of malaria continues to pose a serious health challenge in sub-Saharan Africa [1]. The disease threatens 300–500 million people and kills more than one million annually [2]. It makes substantial demands on Africa's fragile health infrastructure and affects the lives of almost all people [3]. Use of ITNs offers a potential strategy for reducing man-vector contact as well as reducing disease mortality and morbidity rates. They have been proven to offer significant personal protection against malaria infections in areas with drug resistance and insufficient health infrastructure [4]. In Africa, they have been effective in reducing malaria mortality and morbidity, illness episodes and anaemia cases among children [5-9]. In Kenya, an intensive five-year effort by Population Services International (PSI) programme, launched in 2002 dramatically increased malaria awareness and net usage among pregnant women and children less than five years [10]. However, despite this demonstrated efficacy, the choice, use and acceptance of ITNs continue to face major socio-economic and cultural challenges in most malaria endemic areas [11,12]. Given that different communities hold variety of beliefs about the cause and transmission of malaria, these factors are critical and cannot be assumed [13]. Therefore, extensive social science studies are required in a variety of geographical settings in order to tailor control interventions, according to local needs and conditions. This paper reports the findings of a study conducted in an irrigated agroecosystem in Central Kenya with an aim of investigating the local knowledge of malaria and mosquitoes as well as bed net use prior to large scale distribution of long-lasting ITNs through IDRC funded project in four study villages.

## Methods

### Study area

The study was conducted in Mwea Division, Kirinyaga District in Central Kenya located approximately 100 km North East of Nairobi in a riverine plain at an altitude of about 1159 m above sea level. It has a population density of 246 persons per km^2 ^in a total area of 581 Km^2^. The main economic activity is rice growing and horticultural farming. Indigenous cattle are kept mainly for beef and draught power. The study area has two rainfall seasons with the long rains occurring from March to May and the short rains from October to November [14,15]. A previous study in the area recorded a malaria parasite prevalence rate of 24% among children aged below nine years of age and a 30–300 fold increase in malaria vectors [16]. ITNs use and distribution in the area is promoted by the Ministry of Health and other partners like PSI and UNICEF at subsidized prices particularly to pregnant women and children under five years. This is one of the key strategic approach of the National Malaria Control Strategy by the Kenyan Government. Refer to an earlier publications for a further description of the study area [16,17].

### Study design

The study used a combination of qualitative and quantitative ethnographic cross-sectional household survey conducted in April 2005. Mwea Division was conveniently selected amongst other divisions because of its biggest burden of malaria in Kirinyaga District. The four study villages were purposefully selected and a standard sample size of 100 households was randomly selected from each village.

### Data collection

Interviews using structured questionnaires were conducted with household heads or spouses from the selected households in the four study villages. The questions focused on various sub-themes like, socio-demographic characteristics of the respondent and issues concerning the role of mosquitoes in malaria transmission. Other issues focused were perceived benefits of bed net use, reasons for treating bed nets and preferred colour and shape of a treated bed net. Questionnaires were prepared in English and verbally translated into the local language (Kikuyu) during interview time. Each question in the questionnaire was interpreted into the local language and corrections made accordingly before administration. Pre-testing was done in a non-study village and adjustments made accordingly. Two Focus Group Discussions (Men and Women) were held in each village with an aim of gathering descriptive information on bed net ownership and use. Clearance for the study was obtained from Ministry of Health. Questionnaires were administered after explaining the purpose of the study and criteria used to select each respondent. Informed verbal and written consents were obtained from the focus group participants and the household heads. Confidentiality of information was maintained during the whole study.

### Data management and analysis

Data was recorded, entered and processed using the Statistical Package for Social Science (SPSS) version 11.5 for windows, MS Access and MS Excel. Association between dependent and independent variables were measured by use of the Chi-square test.

## Results

### Socio-demographic information of the respondents

At the end of the study, 127 (34.5%) males and 241 (65.5%) females were successfully interviewed. Most of the respondents were protestants (54.9%) followed by catholic (43.8%). On marital status, 68.5% were married, 17.1% windowed, 8.2% single and 6.3% were separated at the time of interview. On occupation, 74.5% of the respondents were farmers, 7.3% were in self business, 4.9% in formal employment and 13.3% in other minor occupations like casual labour. Out of the total respondents, 40.5% had only completed primary education, 18.2% had dropped out at primary school, 13.9% managed to complete secondary level, 3.8% dropped in secondary schools, 19.8% were informally educated and 3.8% with university/college education only.

### Respondents' perception on common illnesses

Malaria was perceived to be a major public health concern and one of the most frequently occurring diseases by 93% of respondents in the four study villages. Typhoid was rated second and bilharzia third by 38% and 10.3% respondents, respectively. There was an observed significant difference in rating the third most frequent occurring disease between the irrigated and non-irrigated villages in the study area. Most respondents in the irrigated villages reported bilharzia as the third most frequently occurring disease, while common cold was perceived to be the third most frequently occurring disease in the non-irrigated villages. The three most frequently mentioned diseases in irrigated areas were all waterborne and this was attributed to presence of large pools of stagnant water in the rice paddies.

### Perceived causes of malaria

Mosquito bite was mentioned to be the main 'cause' of malaria by 95% of the respondents. Other mentioned causes were: long rains or being rained on (12.5%), stagnant water (16%), dirty domestic surroundings (4.6%), wet and cold conditions (10.6%), eating raw food/mangoes (5.2%) and taking of dirty or polluted water (4.1%). Significantly, more males (10.5%) compared to females (2.5%) believed that malaria could also be caused by eating of raw food/mangoes (χ^2 ^= 10.19; df; P = 0.001) (Table 1). Participants' perceptions on the link between malaria and non-biomedical causes were expressed during FGDs in all the four villages:

**Table 1:** Perceived causes of malaria

**Perceived cause**	**χ^2 ^Tests**					
	% Responses (n = 368)	Village	Age	Gender	Education	Occupation
Working in the sun	0.8	0.225	0.911	0.966	0.143	0.227
Long rains/Being rained on	12.5	0.000	0.908	0.709	0.093	0.061
Wet and cold condition	10.6	0.363	0.715	0.870	0.942	0.275
Working in rice paddies	3	0.451	0.174	0.930	0.083	0.255
Mosquito bite	94.6	0.154	0.000	0.914	0.850	0.973
Eating raw foods/mangoes	5.2	0.000	0.804	0.001	0.443	0.204
Evil spirit/Demons/Witchcraft	0.3	0.127	0.981	0.644	0.626	0.953
Taking dirty/Polluted water	4.1	0.081	0.009	0.648	0.210	0.850
From another person with malaria	0.8	0.479	1	0.687	0.805	0.382
Stagnant water	16	0.467	0.532	0.430	0.122	0.113
Dirty home surroundings/Environment	4.6	0.019	0.699	0.689	0.316	0.283
Don't know	1.1	0.532	0.000	0.687	0.135	0.846
Others	2.4	0.001	0.002	0.432	0.404	0.620

*'Fruits like mangoes and tomatoes are bitten by mosquitoes and when people eat them they get malaria' *(Kagio, Women FGD).

*'Mosquitoes bite mangoes and injects the germs and when one eats the mangoes s/he gets malaria' *(Murinduko Women FGD).

*'Some people become sick after eating raw food like tomatoes, mangoes and fermented porridge. I think it is the sourness, which makes the germs to become active in the body' *(Murinduko and Mbui Njeru Men and Women FGDs).

*'Most people in the village don't have good health due to poor nutrition and when they get exposed to cold weather the body become weak and they get sick with malaria' *(Murinduko Women FGD).

### Knowledge of signs and symptoms of malaria

The most common cited signs and symptoms of malaria were headache (70%), feeling cold (65%), (with the tendency to bask in the sun), fever (57%), general body weakness (57%), body/joint pains and vomiting. The level of knowledge on common signs and symptoms of malaria was average in all the villages and there was no significant difference in the score between the villages.

### Reported seasonality of mosquitoes and malaria illness episodes

**Figure 1:**
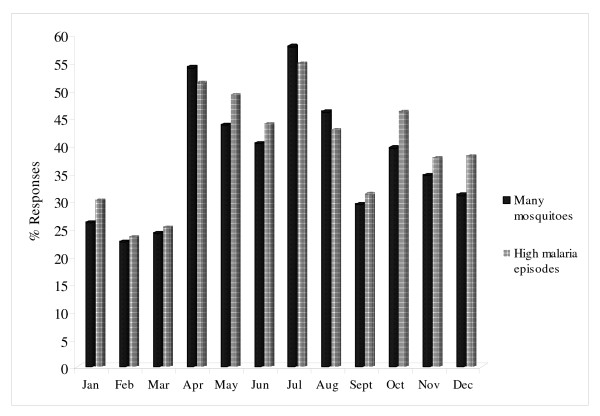
Reported seasonality of mosquitoes and malaria illness episodes.

Overall, most respondents reported that the months of July (57%), April (53%), August (46%) and May (44%) were the four months of the year with high cases of illness episodes perceived to be malaria in the area (Multiple responses). However, all the other months of the year were mentioned but by relatively less number of respondents indicating that the disease was perceived to be endemic in the study area. The same months associated with high illness episodes were reported to experience high numbers of indoor and outdoor nuisance adult biting mosquitoes. This corresponded well with the peak rice growing seasons in the rice irrigated areas and also with long rain season in the non-irrigated areas (Figure 1). Most focus group discussion participants in irrigated areas attributed this to the presence of flooded rice paddies while non-irrigated areas attributed it to the presence of vegetations around houses. The expansion of informal "*Jua Kali*" small scale rice farming outside the scheme was also blamed for the current increases in mosquito numbers and malaria cases in the previously non-irrigated areas.

### Bed net ownership, access and use

Seventy five per cent (75%) of the households reported to own at least one bed net. However, only 48.6% of the total population (1,776) had access to bed nets and 46.7% reported to have slept under bed net in the previous night. This translated into 96% use among the people who had access to bed nets. Among the reported nets, 62% were insectide-treated and the remaining 38% were untreated. There was significant variations between Age of the occupant (χ^2 ^= 60.103; df 7; P = 0.000), village type (χ^2 ^= 143.327; df 3; P = 0.000), relationship to the household head (χ^2 ^= 104.541; df **7**; P = 0.000) and access to bed net among the population. The same variables caused significantly variations on both the conditions of bed nets present and the number of people who slept under bed nets in the previous night. There was a significant variation in the number of bed nets owned between the four study villages (χ^2 ^= 29.4; df 3; P = 0.000), with both Mbui-Njeru (91%) and Kagio (77%) reporting the highest number of households owning at least a bed net during the interview time. There were significance differences in reported use of treated mosquito nets between different socio-demographic profiles of the respondents like occupation (χ^2 ^= 7.955; df 3; P = 0.047), gender (χ^2 ^= 4.254; df 1; P = 0.039), education levels (χ^2 ^= 33.622; df 6; P = 0.000).

### Associated benefits of bed net use

**Figure 2:**
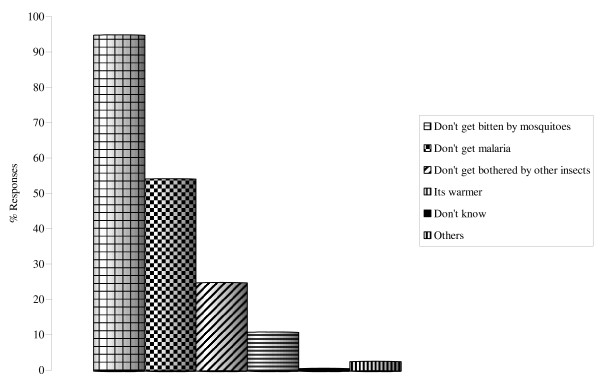
Benefits associated with bed nets use.

**Figure 3:**
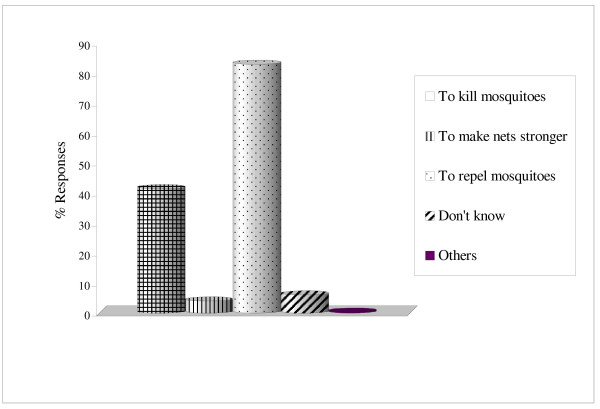
Main reasons for treating bed nets with insecticides.

Among the households owning bed nets, 95% cited protection against mosquito bite as the main reason for using bed nets. Use of bed nets for protection against malaria was mentioned second by 54% (n = 300) of the respondents. Other reported benefits of using bed net were protection from other nuisance insects (25%) while 11% of the respondents acknowledged that the nets offered them warmth at night (Figure 2). A small percentage (13%) cited hotness as one of the major problems associated with sleeping under bed nets especially during the hot months of the year. Other mentioned problems were lack of enough air circulation (3%), difficulty in tacking in the nets each night (3%) and difficulty in getting up at night (2%). Main reason given for treating bed nets with insecticides was to repel the nuisance adult biting mosquitoes at night (83%, n = 350). Other mentioned reasons were to kill mosquitoes (42%) and to make the nets stronger (4%; Figure 3).

### Affordability, shape and colour preference for bed nets

Overall, despite the general feeling from most respondents that treated nets were very important in protecting their households from mosquito bites, 55% of the respondent in the sample, felt that they could only afford to pay for 50 Ksh (0.67 US$) for a medium sized ITN. This was the subsidized price for a medium sized bed net during this survey in a nearby government dispensary (meant for pregnant women and children less than five years of age). Rectangular-shaped bed nets were preferred by 63% of the respondents. However, in Murinduko village, 52% preferred the conical shaped nets, while in Kagio, the differences in preference between rectangular and round/conical-shaped bed nets were not significant. Green-coloured bed nets were reported to be the most preferred colour by most respondents (51%) in the whole study area. Other preferred colours reported were navy blue (21%), white (16%) and light blue (11%).

## Discussion

Malaria was ranked as one of the most frequently occurring disease in Mwea division. The responses from this study corresponded well with the hospital data collected from the nearest sub-district hospital in a previous study in 2001 and 2002 [16]. The study also revealed that Mwea community recognized the role of mosquitoes in malaria transmission and this was confirmed during the focus group discussions in all the villages. However, unlike in other areas in East and West Africa [18,19], the disease was reported to have no vernacular name and was locally referred to as "*mareria*" which is a coined English name for the biomedical term malaria. It was noted that some respondents believed that the disease could be transmitted by other non-biomedical causes such as eating of raw foods (like mangoes, sour porridge) and exposure to environmental conditions such as long rains, wet and cold conditions. Such beliefs can negatively influence the choice, acceptance and utilization of malaria control interventions and consequently impact on treatment seeking, management, prevention and control of the disease [19-22]. The target community may also have difficulties in accepting that mosquito net alone can offer significant protection against malaria. Moreover, bed net users may get disappointed when the signs, symptoms or illness episodes locally perceived to be malaria continues to manifest even after continued adherence to bed net use.

In total, 95% of the respondents in the study area reported to use bed nets mainly for protection against the nuisance adult biting mosquitoes. They valued bed net use mainly for affording them good sleep free from nuisance biting mosquitoes, which were reported to cause trouble by their nuisance biting at night. The link between bed net use and malaria control was only reported second by 54% of the respondents. The use of bed nets by Mwea residents mainly for personal protection against nuisance biting mosquitoes and other nuisance domestic insects can be used as a promotion tool for bed net use. However, this approach can have limitations in that the users may only use bed nets when the mosquito density is high and it may also give the impression that bed nets are simple luxury items and not a priority for malaria control in endemic areas [23,24]. Another health implication of promoting bed net use for protection against nuisance biting mosquitoes is that local people generally view the risk of disease as being directly proportional to mosquito population and during seasons when the numbers of indoor and outdoor nuisance adult biting mosquitoes are low, the rate of bed net access and use may decrease [11,25]. This may put the community at more risk of malaria infection mainly because the relatively perceived low vector density may be efficient enough in infecting people and transmitting the *plasmodium *parasites among the population. Also, people from areas with low annual mosquito densities especially those outside irrigated areas may be less concerned in taking preventive action against the vectors, putting them at a high risk of infection. The combinations of the above social issues could considerably explain the previously reported concept of "paddies paradox" in Mwea where irrigated areas had low prevalence of malaria parasites in the population compared to non-irrigated areas despite having 30–300 folds increase in malaria vectors [16].

It was discovered that most people were unwilling to report problems associated with sleeping under bed nets because it was culturally not good to do so especially when there was some hope of external assistance in the near future. Given that even moderately perceived side effects may cause concern and affect acceptance, compliance and use of treated bed nets, people may need to be informed that the insecticide is safe and minor or temporary side effects could be experienced particularly during the first few days of use [25]. On bed net shape preferences, rectangular-shaped nets were said to accommodate comparatively more people with minimum exposure to mosquito bites. They were preferred because of the sleeping arrangement (bed sharing) common in most households in the study area. Green coloured nets were said to accommodate more dirt compared to other bright coloured nets. Another major reason for preferred shape and colour of bed nets was because of the local housing design and commonly used methods of cooking and lighting. The firewood used for cooking was said to emit a lot of smoke, which made the bright coloured bed nets to look and remain dirty always. Given that most rural households have competing needs and trade offs the cost of bed net was shown to be a relevant factor in determining their widespread ownership and use. Generally, the market price of a medium sized bed net could appear cheap to the outsider but might be unaffordable to most households [26]. Given that the cost could present a significant drain on hardly earned cash in most households, especially if they are also affected by other health and social problems like poor nutrition and inadequate living standards [27]. Findings from this study demonstrated the need for incorporating social, cultural and economic aspects of community during project design and implementation. This would enhance and ensure sustainability of malaria control interventions in developing countries.

## Competing interests

The authors declare that they have no competing interests.

## Authors' contributions

PN conducted the field work, data interpretation and manuscript preparation, GJ, VK and JS provided scientific guidance in data collection, analysis and final manuscript preparation. CK, LK, JG and CM provided guidance in data collection and supervision of the project. All authors have read and approved the final manuscript.
